# Computation-Aided Design of Albumin Affibody-Inserted Antibody Fragment for the Prolonged Serum Half-Life

**DOI:** 10.3390/pharmaceutics14091769

**Published:** 2022-08-24

**Authors:** Na Hyun Kwon, Jae Hun Lee, Inchan Kwon

**Affiliations:** School of Materials Science and Engineering, Gwangju Institute of Science and Technology (GIST), Gwangju 61005, Korea

**Keywords:** single-chain antibody fragment, albumin affibody, half-life extension, fusion protein, structure prediction, cancer

## Abstract

Single-chain variable fragments (scFvs) have been recognized as promising agents in cancer therapy. However, short serum half-life of scFvs often limits clinical application. Fusion to albumin affibody (ABD) is an effective and convenient half-life extension strategy. Although one terminus of scFv is available for fusion of ABD, it is also frequently used for fusion of useful moieties such as small functional proteins, cytokines, or antibodies. Herein, we investigated the internal linker region for ABD fusion instead of terminal region, which was rarely explored before. We constructed two internally ABD-inserted anti-HER2 4D5scFv (4D5-ABD) variants, which have short (4D5-S-ABD) and long (4D5-L-ABD) linker length respectively. The model structures of these 4D5scFv and 4D5-ABD variants predicted using the deep learning-based protein structure prediction program (AlphaFold2) revealed high similarity to either the original 4D5scFv or the ABD structure, implying that the functionality would be retained. Designed 4D5-ABD variants were expressed in the bacterial expression system and characterized. Both 4D5-ABD variants showed anti-HER2 binding affinity comparable with 4D5scFv. Binding affinity of both 4D5-ABD variants against albumin was also comparable. In a pharmacokinetic study in mice, the 4D5-ABD variants showed a significantly prolonged half-life of 34 h, 114 times longer than that of 4D5scFv. In conclusion, we have developed a versatile scFv platform with enhanced pharmacokinetic profiles with an aid of deep learning-based structure prediction.

## 1. Introduction

**Figure 1:**
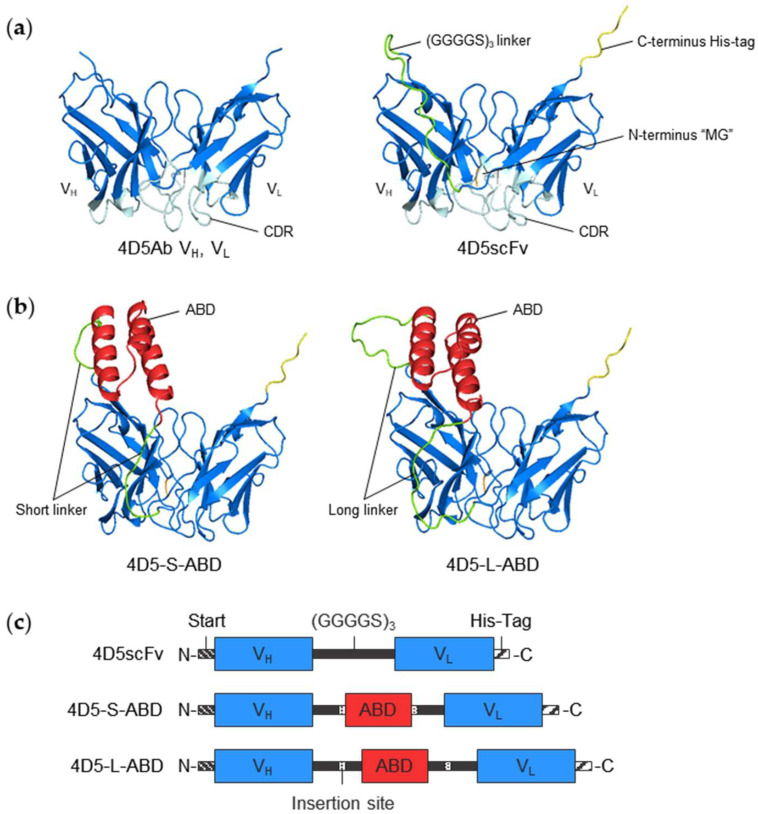
(**a**) Structures of V_H_ and V_L_ region of 4D5Ab (PDB ID:1FVC [14]) and model structure of designed 4D5scFv predicted by AlphaFold2. N-terminus is close to CDR, whereas C-terminus and internal linker are far away from it (**b**) Model structures of two 4D5-ABD variants. Inserted ABD is located away from CDR (blue: V_H_ and V_L_ region; light blue: CDR; green: internal (GGGGS)_3_ linker; orange: “MG” start restriction sequence at N-terminus; yellow: His-tag at C-terminus; red: ABD). All model structures were visualized by PyMOL. (**c**) Schematic diagram of composition of 4D5scFv and two 4D5-ABD variants.

The therapeutic antibody market has grown tremendously since its approval in 1986 [1]. As an alternative to monoclonal antibodies that require costly and laborious mammalian expression systems and suffer from tissue penetration problems, various antibody fragment formats have been investigated [2]. Among antibody derivatives, single-chain variable fragments (scFvs) were first described by Bird et al. in 1988 [3] and several scFv-based therapeutics have been approved by the Food and Drug Administration, demonstrating their clinical potential [4]. An scFv consists of one heavy- and one light-chain variable domain (V_H_ and V_L_, respectively) of immunoglobulins (Igs), connected by a short and flexible polypeptide linker such as (GGGGS)_3_ (Figure 1a) [5,6]. Because scFvs do not contain glycosylation and have only two disulfide bonds in their structure, they can be successfully produced using a bacterial expression system [7]. Furthermore, scFvs do not cause unwanted crystallizable fragment (Fc)-mediated immune responses [8] but have better tumor penetration and distribution than full-length antibodies [9,10]. However, the serum half-lives of scFvs are usually very short because of their small size (approximately 25 kDa) and lack of an Fc region [11,12]. Accordingly, its clinical application was limited to some cases, such as blood cancer or macular degeneration [13]. Therefore, prolonging the serum half-life of scFvs is important for the development of scFv-based therapeutics.

Serum albumin is the most abundant serum protein and has an exceptionally long serum half-life (3 weeks in humans) via neonatal Fc receptor (FcRn)-mediated recycling [15,16,17]. Strategies utilizing albumin, including albumin fusion [18,19,20] or conjugation [21,22,23], are recognized as promising strategies for extending the serum half-life of protein therapeutics. Alternatively, indirect albumin binding using albumin affibodies was investigated to prolong the serum half-life of protein therapeutics [24,25]. The albumin-binding domain (ABD) originating from *Streptococcal* protein G, was developed as a serum half-life extender of its fusion partner through non-covalent binding to serum albumin in vivo [26,27]. ABD fusion has some advantages over other albumination strategies because it employs a microbial expression system and does not require additional purification steps. Therefore, we chose to explore a way to extend the half-life of scFvs via the ABD fusion strategy.

A key factor in the successful design of ABD-fused scFv is the selection of an optimal fusion site to maintain the antigen-binding affinity of scFv and the albumin-binding capacity of ABD. Since one of the two terminals of scFv is close to the complementarity determining region (CDR), the other terminus that is located away from the CDR has been mainly used for scFv fusion studies [28,29,30]. For example, in an scFv configured in V_H_-V_L_ order, N-terminus is close to CDR, so C-terminus fusion is required to avoid interfering antigen binding. However, C-terminus is often required for the fusion of other small functional proteins, cytokines, or antibodies [31,32,33,34]. Instead, we found that the internal linker between V_H_ and V_L_ is an attractive alternative to the terminal for the ABD fusion site because most of the linker region is also apart from the CDR and has no defined structure. To the best of our knowledge, this is an unexplored territory that has never been used for ABD fusion to scFv. Moreover, it was reported that internally inserted ABD retained their binding affinity to albumin [35]. Therefore, we hypothesized that the insertion of ABD into the loop region of scFv will maintain both the antigen-binding affinity of scFv and the albumin-binding affinity of ABD. Most importantly, these constructs will still have at least one terminus available for fusion with other useful proteins, allowing it to function as a versatile half-life extended scFv platform. 

Recently, the development of protein structure prediction methods has been remarkable [36,37]. It reached a crescendo with AlphaFold2, success of which was driven by the revolution of deep learning techniques [38,39]. To verify the structural integrity of the designed protein, we used AlphaFold2 to predict and evaluate the model structure. As a model scFv, we chose 4D5scFv derived from trastuzumab, a monoclonal antibody that recognizes human epidermal growth factor receptor 2 (HER2). Trastuzumab has been used to treat breast cancers overexpressing HER2. We generated two ABD-inserted 4D5scFv variants (4D5-ABD) with different linker lengths (Figure 1b). The structure of the designed molecules was first examined using AlphaFold2, and their binding affinities toward its ligands and pharmacokinetic profiles were analyzed.

## 2. Materials and Methods

### 2.1. Materials

*Escherichia coli* TOP10 competent cells (C404010) were purchased from Invitrogen (Karlsruhe, Germany). Bactotryptone (#211705) and yeast extract (#212750) were obtained from BD Biosciences (San Jose, CA, USA). Nickel-nitrilotriacetic acid (Ni-NTA) agarose resin (#30210) was obtained from Qiagen (Hilden, Germany). Disposable PD-10 desalting columns (#GE17-0851-01) were purchased from Cytiva (Uppsala, Sweden). A steel target plate (#8280781) and Protein Standard II (#8207234) were obtained from Bruker (Billerica, MA, USA). Trifluoroacetic acid (TFA; 99%) was obtained from Daejung Chemicals & Metals (Gyeonggi, Korea). Immunoplates (96-well, flat, #32396) were obtained from SPL Life Sciences (Pocheon, Korea). Recombinant human HER2 (#10004-HCCH) was obtained from Sino Biological (Beijing, China) and Tween-20 (#1610781) was obtained from Bio-Rad (Hercules, CA, USA). Skim milk (#SKI400) was obtained from BioShop (Burlington, ON, Canada). Horseradish peroxidase (HRP)-linked anti-rabbit IgG antibody (#7074) was obtained from Cell Signaling Technology (Danvers, MA, USA). Hydrochloric acid (HCl; #001_00122) was obtained from Duksan Central Science (Seoul, Korea). *L*-(+)-arabinose (#A11921), disposable polypropylene columns (#29924) and Zeba Spin Desalting Columns (7000 molecular weight cut-off (7K MWCO), #89882) were obtained from Thermo Fisher Scientific (Seoul, Korea). Ampicillin (#A0166), sinapic acid (SA; #D7927), human serum albumin (HSA; #A3782), anti-His-tag rabbit antibody (#SAB1306082), and 3,3’,5,5’-Tetramethylbenzidine (TMB; #T4444) were purchased from Sigma-Aldrich (St Louis, MO, USA). All other chemical reagents were purchased from Sigma-Aldrich (St Louis, MO, USA) unless otherwise indicated.

### 2.2. Design and Plasmid Construction

The amino acid sequence of 4D5scFv protein consisted of the N-terminal V_H_ and C-terminal V_L_ regions (PDB ID:1N8Z [40]) linked by a flexible 15-amino acid length (GGGGS)_3_ linker. At the N-terminal, ‘MG’ sequence was incorporated as an *Nco*I restriction site, and at the C-terminal, hexahistidine-tag (His-tag) sequence was incorporated as a protein purification tag. The gene encoding the amino acid sequence was optimized using the ExpOptimizer (NovoPro Bioscience, Shanghai, China), and synthesized and subcloned into *Nco*I/*Kpn*I restriction sites within the pBAD vector by Macrogen (Seoul, South Korea), generating the pBAD_4D5scFv plasmid. The full amino acid and base pair sequence of 4D5scFv are listed in Tables S1 and S2.

The amino acid sequence of one 4D5-ABD variant consisted of an inserted internal ABD sequence within the linker sequence of 4D5scFv. As a *Spe*I restriction site, a ‘TS’ sequence was incorporated at the center of the linker of 4D5scFv and at both ends of the ABD sequence to generate 4D5-S-ABD. For 4D5-L-ABD, an additional linker sequence was inserted between ABD and the restriction site. The gene encoding the ABD sequence was obtained from the plasmid used in the previous study [24], and only the linker sequence was additionally optimized by ExpOptimizer (NovoPro Bioscience). The designed genes for 4D5-ABD variants were synthesized and subcloned into *Afe*I/*Ban*II restriction sites within pBAD_4D5scFv plasmid by Macrogen to generate pBAD_4D5-S-ABD and pBAD_4D5-L-ABD, respectively. The full amino acid and base pair sequences of the two 4D5-ABD are shown in Tables S1 and S2.

### 2.3. Structural Computation

The structures of the designed proteins were evaluated computationally to verify their structural validity. The model structures of the 4D5scFv and 4D5-ABD variants were generated by protein structure prediction using ColabFold [41], a web server that made AlphaFold2 available [38]. The resultant models were evaluated using predicted template modelling (pTM) score and predicted local distance difference test (pLDDT) calculated by AlphaFold2. The structural integrity of the designed protein was analyzed by TM score obtained by alignment with the original protein using the Pairwise Structure Alignment tool of RCSB PDB [42]. The predicted model structure and alignment were visualized using the PyMOL Molecular Graphics System (Version 2.5.2 Incentive Product, Schrödinger, NY, USA).

### 2.4. Expression and Purification

The three plasmids (pBAD_4D5scFv, pBAD_4D5-S-ABD, and pBAD_4D5-L-ABD) were transformed into *Escherichia coli* TOP10 host cells. The transformed cells were cultured at 37 °C for 16 h in standard 2xYT medium containing 100 µg/mL ampicillin while shaking at 200 rpm. The cells were inoculated into the same fresh medium and incubated until the optical density at 600 nm (OD_600_) reached 0.5 to 0.6. Protein expression was induced by *L*-(+)-arabinose at a final concentration of 0.2% (*w*/*v*). After the temperature was lowered to 23 °C, the culture medium was incubated for 24 h. The grown cells were harvested by centrifugation at 8000 rpm and 4 °C, and the cell pellets were stored at −80 °C. Before (BI) and after induction (AI) samples for each variant were collected and centrifuged at 13,000 rpm for 1 min, and then resuspended in PBS (pH 7.4) containing 2 M urea for SDS-PAGE analysis.

His-tagged 4D5scFv and 4D5-ABD variants were purified by metal affinity chromatography using Ni-NTA. The cell pellets were dissolved in lysis buffer (10 mM imidazole, 50 mM NaH_2_PO_4_, 300 mM NaCl, pH 8.0) with 1 mg/mL lysozyme and 5 µg/mL DNase and incubated on ice for at least 5 min. The solution was sonicated at 1 s pulse with 2 s rest and 28% amp (500 W, 20 kHz) for a total of 15 min, which was repeated after 5 min of rest. The mixture was then centrifuged at 10,000 × *g* at 4 °C for 20 min, and the supernatant was incubated at 4 °C for 30 min with Ni-NTA agarose resin. The incubated resin was loaded onto a polypropylene column with a filter before being washed with wash buffer (20 mM imidazole, 50 mM NaH_2_PO_4_, 300 mM NaCl, pH 8.0) and eluted with elution buffer (250 mM imidazole, 50 mM NaH_2_PO_4_, 300 mM NaCl, pH 8.0). The buffer was exchanged with PBS (pH 7.4) using a PD-10 desalting column, according to the manufacturer’s protocol. The purified proteins were stored at 4 °C and analyzed by SDS-PAGE.

### 2.5. Sodium Dodecyl Sulfate Polyacrylamide Gel Electrophoresis (SDS-PAGE) Analysis

The prepared proteins, including resuspended BI and AI samples and purified proteins, were analyzed using SDS-PAGE. All the samples were mixed with 2× sample loading dye (0.2% bromophenol blue, 4% SDS, 20% glycerol, 100 mM Tris-HCl, pH 6.8) containing 100 mM dithiothreitol (DTT) for reduction. For the preparation of non-reducing samples, DTT was excluded. The samples were subjected to 12% SDS-PAGE gel after boiling at 100 °C for 10 min. After electrophoresis, the gels were stained with Coomassie brilliant blue (0.25% Coomassie blue, 50% ethanol, 10% acetic acid) and destained (50% ethanol, 10% acetic acid). The gels were imaged and visualized using ChemiDoc XRS+ System (Bio-Rad, Hercules, CA, USA).

### 2.6. Matrix-Assisted Laser Desorption/Ionization Time-of-Flight (MALDI-TOF) Analysis

The purified samples in PBS (pH 7.4) were desalted using Zeba Spin Desalting Column (7K MWCO) and prepared in 0.1% TFA solution. The samples were mixed with the SA matrix saturated in TA30 (30% acetonitrile, 0.1% TFA) solution at a ratio of 1:1 (*v*/*v*). The SA matrix saturated in ethanol solution was pre-deposited on the steel target plate, and then the sample mixture solutions were deposited above the layer to perform molecular weight analysis using Autoflex speed (Bruker, Billerica, MA, USA). Protein Standard II was used as a calibration standard.

### 2.7. Size Exclusion Chromatography (SEC)

The purified samples were characterized by fast protein liquid chromatography SEC. Equimolar amounts (5 µM) of samples prepared in PBS (pH 7.4) were injected to Superdex 75 Increase 10/300 GL column (Cytiva, Uppsala, Sweden) and eluted at a flow rate of 0.2 mL/min. Ovalbumin, conalbumin, and aldolase from Gel Filtration Calibration Kit HMW (Cytiva, Uppsala, Sweden) were used as standard proteins. The analysis was performed using of NGC Quest 10 Chromatography System (Bio-Rad, Hercules, CA, USA).

### 2.8. Enzyme-Linked Immunosorbent Assay (ELISA)

Anti-HER2 targeting efficiency of purified 4D5scFv variants was analyzed by anti-HER2 ELISA in the presence or absence of HSA. Recombinant HER2 antigen (500 pg/µL, 100 µL/well) in a coating buffer (PBS, pH 7.4) was coated onto the immunoplate by overnight incubation at 4 °C. The plate was washed three times by shaking with PBST (0.05% Tween 20) at 200 µL/well. Then, the plate was blocked by incubating with the blocking buffer (5% skim milk in PBST) at 200 µL/well at room temperature and subsequently washed four times. The purified protein was incubated in PBS (pH 7.4) with or without HSA at a 1:2 molar ratio at room temperature. The incubated protein was prepared in blocking buffer to make a 50 nM stock, which was serially diluted threefold. Prepared samples (100 µL/well, *n* = 2) were loaded onto a plate and incubated at room temperature, and the unbound protein was washed four times. The plate was incubated with 100 µL/well of rabbit anti-His-tag antibody diluted 1:1500 in blocking buffer. After incubation, the wells were washed four times. Finally, the plate was incubated with 100 µL/well of HRP-linked anti-rabbit IgG antibody diluted 1:3000 in blocking buffer. The wells were washed four times. Detection was conducted by adding 100 µL/well TMB, and the reaction was quenched with 2 M HCl. The bound proteins were quantified by measuring absorbance at 450 nm.

The anti-HSA efficiency of the purified 4D5-ABD variants was also investigated by ELISA. HSA (500 pg/µL) in the coating buffer was coated onto the plates. Purified proteins were prepared at 20 nM in blocking buffer and serially diluted threefold. The other conditions and procedures were performed as described above.

### 2.9. Pharmacokinetic Study

Animal studies were performed in accordance with the guidelines of the Animal Care and Use Committee of the Gwangju Institute of Science and Technology (GIST). The serum half-lives of the 4D5scFv variants were investigated using mouse experiments. Purified 4D5scFv and 4D5-ABD variants (100 µg/mL) prepared in 200 µL PBS (pH 7.4) were injected into the tail vein of 9-week-old female BALB/c mice (*n* = 4). Blood samples (<100 µL) were collected at 3 min and 1, 2, 4, 8, 24, and 48 h after injection through retro-orbital blood collection. The serum was separated from the blood by centrifugation at 10,000 × *g* at 4 °C for 10 min and was stored at −20 °C until further use. The serum concentration of protein at each time point was determined by ELISA, as described above. The concentrations were calculated by interpolating the standard calibration curves.

## 3. Results and Discussion

### 3.1. Protein Design and Computation

The ABD-free 4D5scFv and ABD-inserted 4D5scFv variants were designed and their structures were predicted by computational simulation. First of all, we first generated 4D5scFv, which consists of the V_H_ and V_L_ regions of the corresponding IgG (4D5Ab), joined together by a flexible (GGGGS)_3_ linker (Figure 1a,c) [3,40]. We located V_H_ region at the N-terminus and V_L_ region at the C-terminus, which are reported to have a higher binding affinity than the opposite [43]. Because the N-terminus is close to the CDR of 4D5scFv, as described in Figure 1a, His-tag was incorporated into the C-terminus to avoid disrupting the binding affinity of 4D5scFv.

The structure of ABD-free 4D5scFv was predicted by AlphaFold2 using the ColabFold web server (Figure 1a) [38,41]. The resulting model structure was evaluated and aligned with original V_H_ and V_L_ regions of 4D5Ab (PDB ID: 1FVC [14]) (see Supplementary Materials for further details). It revealed that the designed 4D5scFv maintained its original structure from 4D5Ab to a considerable extent; therefore, it can be predicted that its anti-HER2 binding affinity, will be maintained.

Subsequently, we generated 4D5-ABD variants with an inserted ABD sequence at the center of the internal linker of 4D5scFv (Figure 1b,c). Two variants, 4D5-S-ABD with no additional linker sequence (short linker) and 4D5-L-ABD with an additional linker sequence (long linker), were designed to determine whether the linker length can affect the functionality of ABD or 4D5scFv in the generated 4D5-ABD variants. The two variants differed in length by nine amino acids. For the insertion of ABD, the SpeI restriction site which encodes amino acid residues “TS” was used, because these residues are similar to linker residues which consists of “G” and “S”.

To verify the structural integrity of these unfamiliar forms of 4D5-ABD variants, their structures were also computationally predicted using AlphaFold2 (Figure 1b) and analyzed by pTM score and pLDDT (Figure S1, Table S3). The pTM scores of 4D5-S-ABD and 4D5-L-ABD were calculated to be 0.76 and 0.75, respectively. The mean pLDDT was calculated for whole, V_H_/V_L_ region only, and ABD region only. The results differed markedly by region: 89.6, 94.7, and 83.1 for 4D5-S-ABD, and 88.7, 95.3, and 84.8 for 4D5-L-ABD, respectively. Notably, the linker and ABD region confidence were relatively low. This is because AlphaFold2 usually poorly describes the behavior of the unstructured region such as linkers or loops [44,45]. Afterwards, the model structures of 4D5-ABD variants were aligned with model structures of 4D5scFv and ABD (Figure S2, Table S4). The TM scores for all alignments were very high, scoring more than 0.94. In other words, structures of 4D5scFv and ABD in the constructed 4D5-ABD variants were highly retained, implying that their functionality will be also retained. Additionally, the predicted model structure showed that the location of inserted ABD is away from CDR of 4D5scFv, as expected. This fact can also be seen in the visualization of 4D5-ABD and HER2 complex (Figure S3), where ABD is clearly separated from the HER2 binding site. Therefore, it can be predicted that the antigen binding affinity of 4D5scFv will not be significantly interfered with by the presence of ABD or albumin binding to ABD. In conclusion, the high structural consistency and positional independence of each component will result in maintaining the functionality of both 4D5scFv and ABD to a large extent.

### 3.2. Expression and Purification

**Figure 2:**
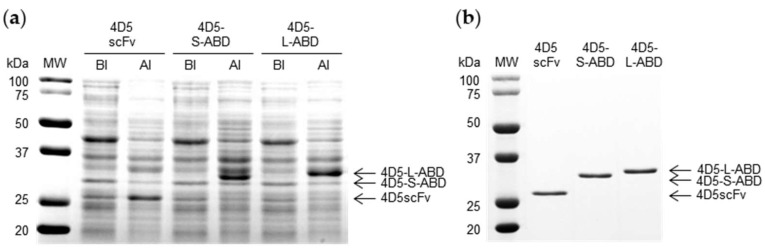
SDS-PAGE analysis of expression and purification of 4D5scFv and two 4D5-ABD variants. (**a**) Coomassie blue-stained SDS-PAGE gel of cell lysate samples attained during expression (MW: molecular weight standards; BI: cell lysate sample before induction; AI: cell lysate sample after induction). (**b**) Coomassie blue-stained SDS-PAGE gel of purified samples.

**Figure 3:**
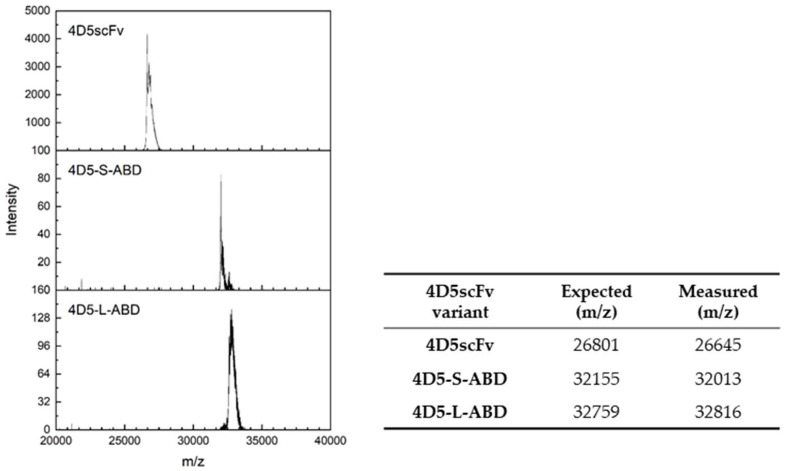
MALDI-TOF analysis of purified 4D5scFv and two 4D5-ABD variants.

Constructed 4D5scFv and two 4D5-ABD variants were produced as a protein by cytoplasmic expression in bacterial host. In the SDS-PAGE analysis of cell lysates collected during expression (Figure 2a), bands were detected in the lanes loaded with AI samples with molecular weights of 27, 32, and 33 kDa, corresponding to those of 4D5scFv, 4D5-S-ABD, and 4D5-L-ABD, respectively. Purification was performed using interaction between Ni-NTA on agarose resin and His-tag affinity tag on C-terminus of each protein. In the SDS-PAGE analysis of the purified proteins, bands at the same position as the expression gel were detected (Figure 2b). In addition, all three proteins showed only monomer protein bands under both reducing and non-reducing condition (Figure S4), indicating that no substantial intermolecular disulfide bonds were formed. Further SEC analysis (Figure S5) showed that 4D5scFv is stable in a mixture of monomers and dimers, which is not formed from disulfide bonds according to non-reducing SDS-PAGE. Conversely, 4D5-ABD variants did not form dimers, but were present exclusively as a monomer, which may be due to steric hindrance generated from fusion of ABD. These three proteins were further identified by MALDI-TOF analysis (Figure 3). In the mass spectra of the intact 4D5scFv, 4D5-S-ABD, and 4D5-L-ABD, peaks were detected at 26,645, 32,013, and 32,816 *m*/*z*. With a deviation less than 1%, these matched well with the expected values of 4D5scFv variants, which were 26,801, 32,155, and 32,759 *m*/*z*, respectively. Production yields of purified 4D5scFv, 4D5-L-ABD, and 4D5-S-ABD were 22.0 ± 0.5, 18.8 ± 1.3 and 21.5 ± 0.8 mg/L, respectively. Overall, these results demonstrate that the 4D5scFv variant samples were successfully expressed and purified.

### 3.3. Binding Affinity Assays

**Figure 4:**
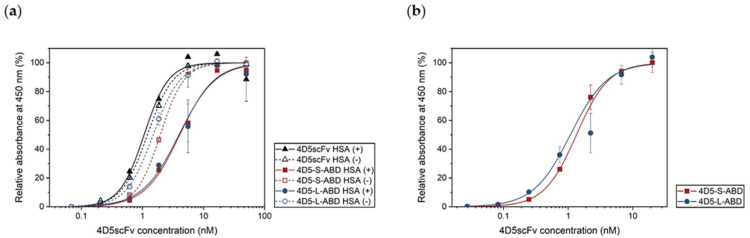
(**a**) Anti-HER2 ELISA of 4D5scFv and two 4D5-ABD variants, in the absence or presence of HSA. (**b**) Anti-HSA ELISA of two 4D5-ABD variants.

The binding affinities of purified 4D5scFv variants against HER2 were studied (Figure 4a, Table 1). All the proteins showed a similar concentration-dependent curve. The half maximal effective concentration (EC_50_) values of 4D5-S-ABD and 4D5-L-ABD were 1.95 and 1.50 nM, respectively, which were very slightly deviated from that of 4D5scFv (1.20 nM). This suggests that ABD insertion did not cause considerable distortion in the structure of 4D5scFv, as expected in the previous computation, and consequently, the anti-HER2 binding affinities of 4D5-ABD variants remained intact.

The binding affinities of 4D5scFv variants in the presence of HSA were also investigated. All the variants were pre-incubated with HSA at a 1:2 molar ratio before ELISA. As a result, the HER2 binding by ABD-free 4D5scFv did not substantially change, showing similar EC_50_ values of 1.06 and 1.20 nM with or without HSA. This indicates that the anti-HER2 binding affinity of 4D5scFv is neither interfered with nor blocked by the presence of HSA. However, 4D5-S-ABD and 4D5-L-ABD variants showed slightly reduced EC_50_ values (4.07 and 3.96 nM, respectively), which are two to three times greater than those without HSA. In other words, HSA binding to 4D5-ABD variants can produce a mild decrease in anti-HER2 binding affinity, likely due to the weak steric hindrance of bulky HSA to HER2 binding with 4D5scFv. Meanwhile, no significant difference in functionality by linker length was observed.

The anti-HSA binding affinities of the 4D5-S-ABD and 4D5-L-ABD variants were also compared (Figure 4b, Table 1). The two variants showed EC_50_ values of 1.34 and 1.08 nM, respectively, where difference between them is negligible considering standard error. It is worth noting that 4D5-S-ABD and 4D5-L-ABD were not distinguished by their binding affinity against either HER2 or HSA, implying that the linker length did not influence the functionality of the variants.

### 3.4. Pharmacokinetic Study

**Figure 5:**
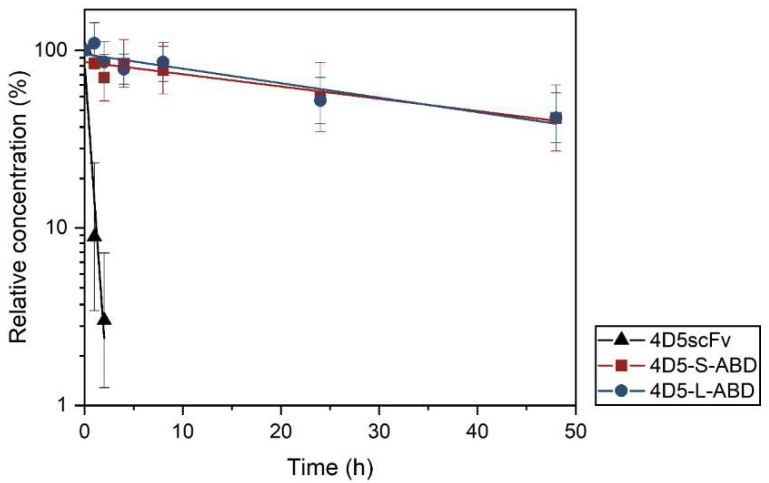
Pharmacokinetic profiles of 4D5scFv and two 4D5-ABD variants.

The serum half-lives of the 4D5scFv and 4D5-ABD variants were determined after intravenous injection to BALB/c mice (*n* = 4). ELISA was performed to quantify the remaining protein in the serum (Figure 5, Table 2). The 4D5scFv showed rapid clearance from the blood with a serum half-life of 18 min, which is consistent with previous research [11,46]. In contrast, 4D5-S-ABD and 4D5-L-ABD exhibited a remarkably extended half-life of 34 h, a 114-fold increase compared to the 4D5scFv. Moreover, both ABD-fused variants showed an approximately 70-fold increase in the AUC values, which were calculated to be 42, 2887, and 3008 for 4D5scFv, 4D5-S-ABD, and 4D5-L-ABD, respectively. Considering that the detection limit has not been reached for 4D5-ABD variants, the actual AUC increase is expected to be much higher.

It can be inferred that the significant increase in half-life in 4D5-ABD variants is because that albumin-bound 4D5-ABD molecules underwent FcRn-mediated recycling. Additionally, the hydrodynamic size increased by albumin binding can be another reason, because it can result in resistance to renal filtration. The result also demonstrates that internally inserted ABD retained its binding affinity to serum albumin, which is consistent with the structural computation and ELISA results. Meanwhile, no difference was observed between the 4D5-ABD variants, which is consistent with the previous ELISA results. This suggests that the linker length of 15 amino acid residues of 4D5-S-ABD variant is sufficient to freely move the ABD moiety to bind to albumin.

## 4. Conclusions

The concepts of internally albumin affibody-inserted scFvs can be of great help in the development of anticancer therapeutics. First of all, an extended half-life is a powerful advantage of expanding the scope of cancer therapy using antibody fragments. In addition, one-step, easy expression of ABD-fused proteins in microbial system can simplify the drug production process. Last but not least, the unmodified terminal site allows for additional fusion of useful partners which may benefit from targeted therapy. For example, many cytokines, including interleukin (IL), tumor necrosis factor (TNF) and interferon (IFN), have been studied to generate payload immunocytokines by fusing them to antibodies or antibody fragments [33]. On the other hand, attention was drawn to the development of bispecific scFv, such as bispecific T cell engager (BiTE), which is a series fusion of two different scFvs [34]. In further research, these cytokines or other antibodies may be fused to the terminal of 4D5-ABD to be developed as therapeutics for breast cancer.

In designing 4D5-ABD variants, we were able to easily generate the model structures via AlphaFold2 and obtain lots of insight from them, proving the usefulness of artificial intelligence in developing therapeutic fusion protein. In addition, other tools such as RoseTTAFold [47] and Rosetta [48], are also greatly benefiting protein engineers. We expect further studies on the fusion of cytokines or antibodies with 4D5-ABD variants can be conducted with the help of such software.

Nevertheless, for 4D5-ABD research to lead to actual drug development, complementary experiments on in vivo distribution are essential. Because small molecular weight of scFv is an important factor for superior distribution compared to monoclonal antibody, influence of increased hydrodynamic size which is resulted from albumin binding cannot be ignored. However, it is known that albumin itself shows preferential uptake toward tumors [49]. Furthermore, it was reported that HSA fusion to scFv increases the tissue penetrability and distribution [50]. Therefore, despite the increased hydrodynamic size of scFv, it can be expected that in vivo distribution will be enhanced compared to unmodified scFvs or monoclonal antibody, due to increased blood circulation time and albumin’s own selectivity toward tumor.

In summary, we demonstrated that 4D5-ABD variants have considerably enhanced half-life, while maintaining functionality of each component. Furthermore, they can be a novel platform which enables the fusion of other useful molecules. Therefore, internally albumin affibody-inserted scFv is a promising and versatile targeting agent that can be used in various clinical applications.

## Figures and Tables

**Table 1:** EC_50_ value of 4D5scFv and two 4D5-ABD variants measured in the anti-HER2 ELISA and anti-HSA ELISA.

4D5scFv Variant	EC_50_ (nM)		
	Anti-HER2 HSA (−)	Anti-HER2 HSA (+)	Anti-HSA
4D5scFv	1.20 ± 0.05	1.06 ± 0.21	-
4D5-S-ABD	1.95 ± 0.05	4.07 ± 0.54	1.34 ± 0.05
4D5-L-ABD	1.50 ± 0.06	3.96 ± 0.98	1.08 ± 0.22

**Table 2:** Pharmacokinetic parameters of 4D5scFv and two 4D5-ABD variants.

4D5scFv Variant	t_1/2_ (h)	AUC (%∙h)
4D5scFv	0.30 ± 0.25	42 ^1^
4D5-S-ABD	34.19 ± 0.05	2887 ^2^
4D5-L-ABD	34.29 ± 0.05	3008 ^2^

^1^ 0–2 h; ^2^ 0–48 h.

## Data Availability

The supporting data presented in this study are available in Supplementary Materials.

## Supplementary Materials

The following supporting information can be downloaded at: https://www.mdpi.com/article/10.3390/pharmaceutics14091769/s1, Figure S1: Computational evaluation of structure prediction of 4D5scFv and two 4D5-ABD variants. (**a**) Model structures predicted by AlphaFold2 and visualized by PyMOL. (**b**) pLDDT per position for structure prediction, reported by AlphaFold2.; Figure S2: Structure alignment of 4D5scFv and two 4D5-ABD variants against original structure using Pairwise Structure Alignment tool. (**a**) Structure alignment of 4D5scFv with V_H_ and V_L_ region of 4D5Ab. (**b**) Structure alignment of 4D5-ABD variants with 4D5scFv. (**c**) Structure alignment of 4D5-ABD variants with ABD.; Figure S3: Structures of complex form of two 4D5-ABD variants and HER2 visualized by PyMOL.; Figure S4: SDS-PAGE analysis of 4D5scFv and two 4D5-ABD variants under reducing (**left**) and non-reducing condition (**right**).; Figure S5: Size exclusion chromatograms of 4D5scFv, two 4D5-ABD variants, and standard proteins.; Table S1: Full amino acid sequence of 4D5scFv and two 4D5-ABD variants.; Table S2: Full base pair sequence of 4D5scFv and two 4D5-ABD variants.; Table S3: pTM score and mean pLDDT of predicted structure of 4D5scFv and two 4D5-ABD variants.; Table S4: The TM score of all the alignment.
