# Supplementary material for: Computation-Aided Design of Albumin Affibody-Inserted Antibody Fragment for the Prolonged Serum Half-Life
Source: Pharmaceutics. 2022 Aug 24;14(9):1769. doi: 10.3390/pharmaceutics14091769 (PMC9500697; doi:10.3390/pharmaceutics14091769)
Supplement: Supplementary file 1 [file pharmaceutics-14-01769-s001.zip › pharmaceutics-1862905-supplementary.pdf]

Supplementary Materials: Computation-Aided Design of Albumin Affibody-Inserted Antibody Fragment for the Prolonged Serum Half-Life

Na Hyun Kwon, Jae Hun Lee and Inchan Kwon

Table S1. Full amino acid sequence of 4D5scFv and two 4D5-ABD variants. V_H and V_L regions were colored blue and ABD red. In Table 4. D5-ABD variants, only linker and ABD sequences were shown. Restriction sites at the start and *Spe*I restriction sites were underlined.

4D5scFv variant	Amino acid sequence
4D5scFv	MGEVQLVESGGGLVQPGGSLRLSCAASGFNIKDTYIHWVRQ APGKGLEWVARIYPTNGYTRYADSVKGRFTISADTSKNTAYLQ MNSLRAEDTAVYYCSRWGGDGFYAMDYWGQGTLVTVSS GGGGS GGGGS GGGGS DIQMTQSPSSLSASVGDRVTITCRASQDVNTAVAWYQQKPGK APKLLIYSASFLYSGVPSRFSGSRSGTDFTLTISSLQPEDFATYYCQ QHYTTPPTFGQGTKVEIKHHHHHH
4D5-S-ABD	GGGGS GTS LAEAKVLANRELDKYGVSDFYKRLINKAKTVEGVEALKLHILAALP TSGS GGGGS
4D5-L-ABD	GGGGS GTSGS GGS LAEAKVLANRELDKYGVSDFYKRLINKAKTVEGVEALKLHILAALP GGS GTSGS GGGGS

Table S2. Full base pair sequence of 4D5scFv and two 4D5-ABD variants. V_H and V_L regions were colored blue and ABD red. In the case of 4D5-ABD variants, only linker and ABD sequences were shown. Restriction sites at the start and end and *Spe*I restriction sites were underlined.

4D5scFv variant	Base pair sequence
4D5scFv	CCATG(Start)GGTGAAGTTCAGCTGGTTGAATCTGGCGGTGGTCTGG<br>TTCAGCCGGGTGGTTCTCTGCGTCTGAGCTGTGCTGCTTCCGGTTTTA<br>ACATTAAAGATACTTACATTCACTGGGTACGTCAGGCCCCGGGTAA<br>AGGTCTGGAATGGGTTGCTCGCATCTACCCGACTAACGGCTACACC<br>CGTTACGCGGACTCTGTCAAAGGTCGTTTTACTATTAGCGCTGATAC<br>CAGCAAAAACACGGCGTACCTGCAAATGAACTCCCTGCGTGCAGA<br>AGATACCGCTGTCTACTACTGTAGCCGTTGGGGCGGTGACGGTTTTT<br>ACGCTATGGACTATTGGGGTCAGGGCACCCTGGTAACCGTATCTTCT<br>GGTGGTGGTGGTTCTGGTGGTGGTGGTTCTGGTGGTGGTGGCTCC<br>GACATCCAGATGACCCAGAGCCCGTCCTCTCTGTCTGCATCTGTTGG<br>TGACCGTGTCACCATTACTTGCCGCGCCTCTCAGGATGTAAATACCG<br>CTGTTGCGTGGTATCAACAGAAACCGGGCAAAGCTCCAAAGCTGCT<br>GATTTACTCTGCTTCTTTCCTGTACTCTGGTGTTCCGTCTCGTTTCTCC<br>GGTTCCCGTAGCGGCACTGACTTTACCCTGACCATCAGCAGCCTGCA<br>ACCGGAGGACTTCGCAACCTACTACTGCCAGCAGCACTACACTACC<br>CCGCCGACCTTCGGTCAGGGTACTAAAGTGGAAATTAAGCACCATC<br>ACCACCACCATTAA(Stop)GGTACC
4D5-S-ABD	GGTGGTGGTGGTTCTGGTACTAGT<br>CTGGCGGAGGCTAAAGTTCTCGCTAACCGCGAACTTGACAAATATGG<br>TGTGAGCGATTTTTACAAGCGTTTAATTAATAAAGCAAAGACCGTCG<br>AAGGCGTAGAGGCATTGAAATTACATATCCTGGCCGCGCTGCCG<br>ACTAGTGGTTCTGGTGGCGGTGGTAGC
4D5-L-ABD	GGTGGTGGTGGTTCTGGTACTAGTGGTTCTGGTGGTTCT<br>CTGGCGGAGGCTAAAGTTCTCGCTAACCGCGAACTTGACAAATATG<br>GTGTGAGCGATTTTTACAAGCGTTTAATTAATAAAGCAAAGACCGTC<br>GAAGGCGTAGAGGCATTGAAATTACATATCCTGGCCGCGCTGCCG<br>GGTGGTTCTGGCACTAGTGGTTCTGGTGGTGGTGGTTCC

Structure prediction of ABD-free 4D5scFv

The structure of ABD-free 4D5scFv was predicted by AlphaFold2 (Figure S1a) [1,2]. The resulting model structure was evaluated by pLDDT and pTM score (Figure S1b and Table S3). Five model structures were generated and ranked by the mean pLDDT value. The top-ranked model structure exhibited a fairly high pTM score of 0.88 and a mean pLDDT of 91.7, suggesting that the generated model structure is reliable. Notably, pLDDT was higher at the VH/VL region than linker region, indicating that regions with defined structures were predicted with a high degree of confidence while linker region was not. This is because AlphaFold2 usually poorly describes the behavior of the unstructured region such as linkers or loops [3,4]. The top-ranked model of 4D5scFv was then aligned with the original VH and VL regions of 4D5Ab (PDB 1D:1FVC [5]) using the Pairwise Structure Alignment tool (Figure S2a, Table S4) [6]. The calculated TM score for alignment of 4D5scFv with VH and VL were 0.98 and 0.99, respectively, indicating that 4D5scFv retained original VH and VL region at a high level.

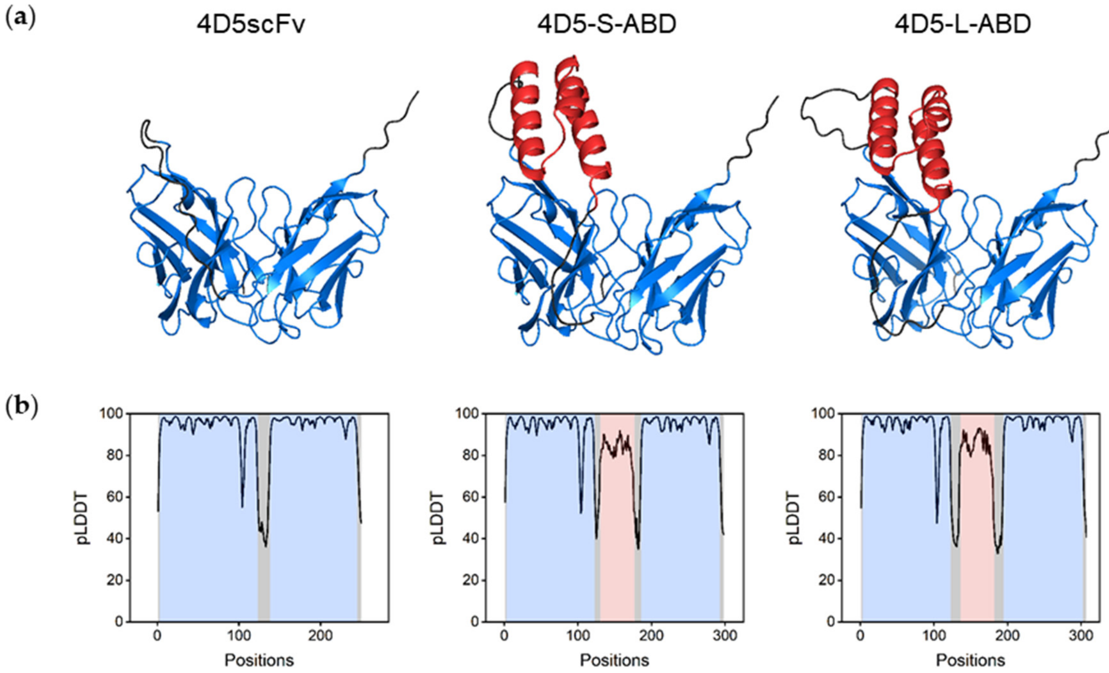

Figure S1. Computational evaluation of structure prediction of 4D5scFv and two 4D5-ABD variants. (**a**) Model structures predicted by AlphaFold2 and visualized by PyMOL. (**b**) pLDDT per position for structure prediction, reported by AlphaFold2. High pLDDT values were observed mainly at the V_H and V_L region which have defined structure. Other regions, which have undefined structures, linker, and loops, exhibited lower values (blue: V_H and V_H region; red: ABD; black: others).

Table S3. pTM score and mean pLDDT of predicted structure of 4D5scFv and two 4D5-ABD variants. Mean pLDDT values were calculated for "whole", "V_H/V_L only" and "ABD only", respectively. Mean pLDDT of "V_H/V_L only" is higher than that of "whole", whereas mean pLDDT of "ABD only" is lower. This is explained that AlphaFold2 generally poorly describes the structure of the unstructured region.

Score	4D5scFv	4D5-S-ABD	4D5-L-ABD
pTM score	0.88	0.76	0.75
Mean pLDDT (whole)	91.7	89.6	88.7
Mean pLDDT (V_H/V_L only)	95.6	94.7	95.3
Mean pLDDT (ABD only)	-	83.1	84.8

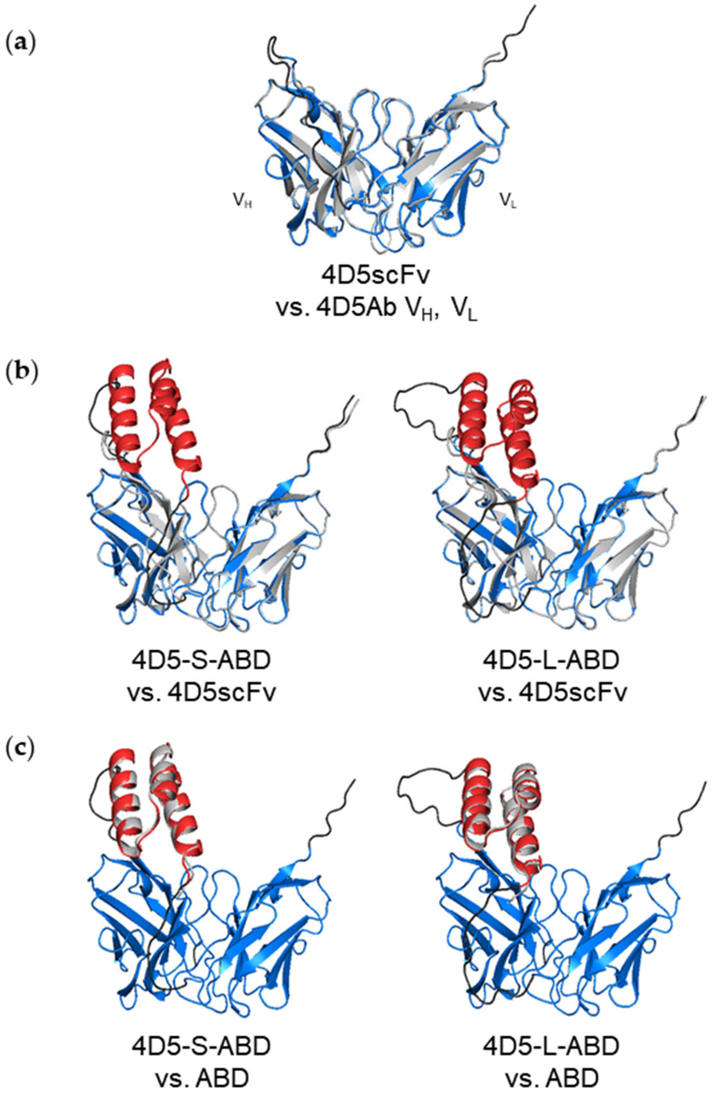

Figure S2. Structure alignment of 4D5scFv and two 4D5-ABD variants against original structure using Pairwise Structure Alignment tool. All images were visualized by PyMOL. (**a**) Structure alignment of 4D5scFv with V_H and V_L region of 4D5Ab (grey, PDB ID:1FVC [5]). (**b**) Structure alignment of 4D5-ABD variants with 4D5scFv (grey). (**c**) Structure alignment of 4D5-ABD variants with ABD (grey) (blue: V_H and V_L region; red: ABD; black: other regions, including start restriction site, linker, His-tag).

Table S4. The TM score of all the alignment.

**Score**	**4D5scFv**
4D5scFv vs. 4D5Ab V_H	0.98
4D5scFv vs. 4D5Ab V_L	0.99
4D5-S-ABD vs. 4D5scFv	0.97
4D5-L-ABD vs. 4D5scFv	0.95
4D5-S-ABD vs. ABD	0.95
4D5-L-ABD vs. ABD	0.94

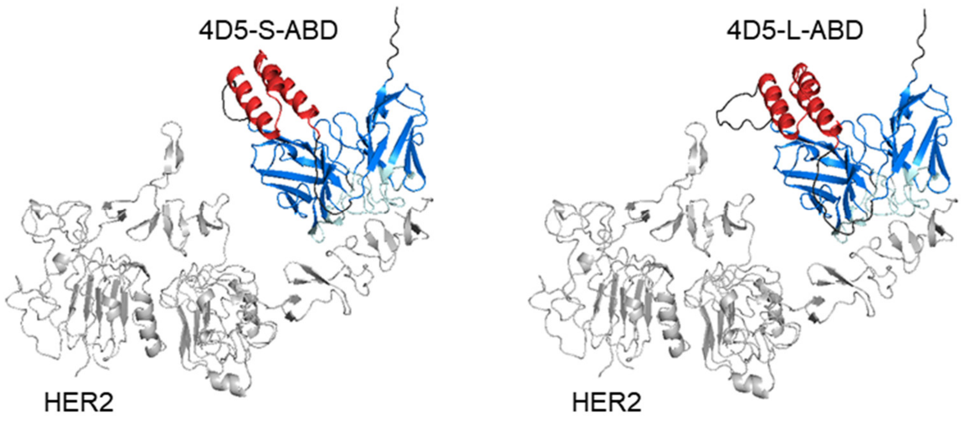

Figure S3. Structures of complex form of two 4D5-ABD variants and HER2 (PDB ID:1N8Z [7]) visualized by PyMOL (blue: V_H and V_L region; red: ABD; light blue: CDR; grey: HER2; black: other regions, including start restriction site, linker, His-tag). Because the internally fused ABD is far from the CDR, it is expected that the effect on antigen binding affinity will not be considerable.

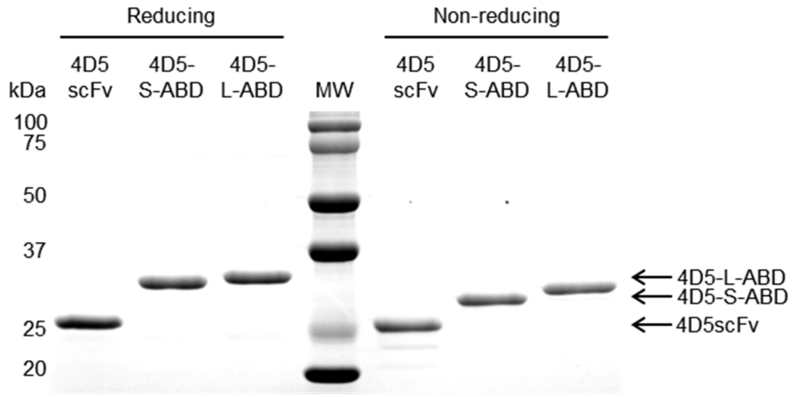

Figure S4. SDS-PAGE analysis of 4D5scFv and two 4D5-ABD variants under reducing (left) and non-reducing condition (right) (MW: molecular weight standards).

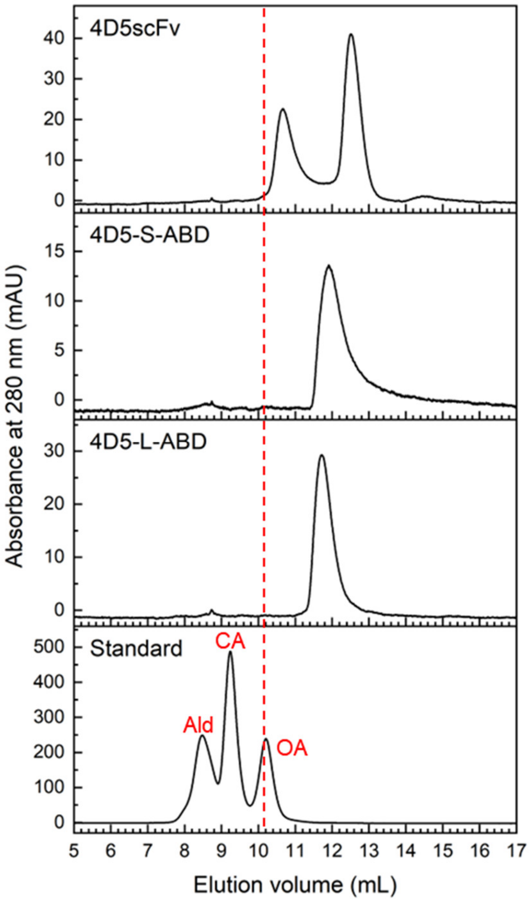

Figure S5. Size exclusion chromatograms of 4D5scFv, two 4D5-ABD variants, and standard proteins (4D5scFv: 27 kDa; 4D5scFv dimer: 54 kDa; 4D5-S-ABD: 32 kDa; 4D5-L-ABD: 33 kDa; ovalbumin (OA): 43 kDa; conalbumin (CA): 75 kDa; aldolase (Ald): 158 kDa).

References

1. Jumper, J.; Evans, R.; Pritzel, A.; Green, T.; Figurnov, M.; Ronneberger, O.; Tunyasuvunakool, K.; Bates, R.; Žídek, A.; Potapenko, A.; et al. Highly Accurate Protein Structure Prediction with AlphaFold. *Nature* **2021**, *596*, 583–589, doi:10.1038/s41586-021-03819-2.
2. Mirdita, M.; Schütze, K.; Moriwaki, Y.; Heo, L. ColabFold: Making Protein Folding Accessible to All. *Nat. Methods* **2022**, *19*, 679–682, doi:10.1038/s41592-022-01488-1.
3. Goulet, A.; Cambillau, C. Present Impact of AlphaFold2 Revolution on Structural Biology, and an Illustration With the Structure Prediction of the Bacteriophage J-1 Host Adhesion Device. *Front. Mol. Biosci.* **2022**, *9*, 907452, doi:10.3389/fmolb.2022.907452.
4. Bouatta, N.; Sorger, P.; AlQuraishi, M. Protein Structure Prediction by AlphaFold2: Are Attention and Symmetries All You Need? *Acta Crystallogr. Sect. D, Struct. Biol.* **2021**, *77*, 982–991, doi:10.1107/S2059798321007531.
5. Eigenbrot, C.; Randal, M.; Presta, L.; Carter, P.; Kossiakoff, A. X-Ray Structures of the Antigen-Binding Domains from Three Variants of Humanized Anti-P185HER2 Antibody 4D5 and Comparison with Molecular Modeling. *J. Mol. Biol.* **1993**, *229*, 969–995, doi:10.1006/jmbi.1993.1099.
6. Burley, S.K.; Bhikadiya, C.; Bi, C.; Bittrich, S.; Chen, L.; Crichlow, G. V.; Christie, C.H.; Dalenberg, K.; Di Costanzo, L.; Duarte, J.M.; et al. RCSB Protein Data Bank: Powerful New Tools for Exploring 3D Structures of Biological Macromolecules for Basic and Applied Research and Education in Fundamental Biology, Biomedicine, Biotechnology, Bioengineering and Energy Sciences. *Nucleic Acids Res.* **2021**, *49*, D437–D451, doi:10.1093/nar/gkaa1038.
7. Cho, H.S.; Mason, K.; Ramyar, K.X.; Stanley, A.M.; Gabelli, S.B.; Denney, D.W.; Leahy, D.J. Structure of the Extracellular Region of HER2 Alone and in Complex with the Herceptin Fab. *Nature* **2003**, *421*, 756–760, doi:10.1038/nature01392.
